# Impaired ADAMTS9 secretion: A potential mechanism for eye defects in Peters Plus Syndrome

**DOI:** 10.1038/srep33974

**Published:** 2016-09-30

**Authors:** Johanne Dubail, Deepika Vasudevan, Lauren W. Wang, Sarah E. Earp, Michael W. Jenkins, Robert S. Haltiwanger, Suneel S. Apte

**Affiliations:** 1 Department of Biomedical Engineering, Cleveland Clinic Lerner Research Institute, 9500 Euclid Avenue, Cleveland, OH 44195, USA; 2 Department of Biochemistry and Cell Biology, Stony Brook University, NY 11794, USA; 3 Department of Pediatrics and Biomedical Engineering, Case Western Reserve University, 11000 Euclid Avenue, Cleveland, OH 44106, USA

## Abstract

Peters Plus syndrome (PPS), a congenital disorder of glycosylation, results from recessive mutations affecting the glucosyltransferase B3GLCT, leading to congenital corneal opacity and diverse extra-ocular manifestations. Together with the fucosyltransferase POFUT2, B3GLCT adds Glucoseβ1-3Fucose disaccharide to a consensus sequence in thrombospondin type 1 repeats (TSRs) of several proteins. Which of these target proteins is functionally compromised in PPS is unknown. We report here that haploinsufficiency of murine *Adamts9,* encoding a secreted metalloproteinase with 15 TSRs, leads to congenital corneal opacity and Peters anomaly (persistent lens-cornea adhesion), which is a hallmark of PPS. Mass spectrometry of recombinant ADAMTS9 showed that 9 of 12 TSRs with the *O*-fucosylation consensus sequence carried the Glucoseβ1-3Fucose disaccharide and *B3GLCT* knockdown reduced ADAMTS9 secretion in HEK293F cells. Together, the genetic and biochemical findings imply a dosage-dependent role for ADAMTS9 in ocular morphogenesis. Reduced secretion of ADAMTS9 in the absence of B3GLCT is proposed as a mechanism of Peters anomaly in PPS. The functional link between ADAMTS9 and B3GLCT established here also provides credence to their recently reported association with age-related macular degeneration.

Anterior segment dysgenesis (ASD) encompasses a group of congenital eye disorders which are an important cause of severe visual loss in children. Typically, one or more ocular anterior segment structures, such as the cornea, lens, iris, ciliary body, and/or aqueous humor drainage apparatus are malformed. The observed congenital anomalies can include corneal opacity, iris hypoplasia, developmental cataract, and adhesions between the iris, cornea and lens, occurring alone or in combination^1^. Mild ASD with abnormalities in the aqueous humor drainage structures can result in glaucoma and subsequent visual impairment^2^. *FOXC1, PITX2, PITX3, FOXE3, PAX6, CYP1B1*, and *COL4A1* mutations are known causes of ASD^3^,^4^,^5^,^6^, and these genes also participate in ocular morphogenesis in mice^7^,^8^,^9^,^10^,^11^,^12^,^13^, but they do not account for all genetic forms of ASD.

A canonical subtype of ASD, named Peters anomaly, constituting corneal opacity, defects in the posterior layers of the cornea, and lenticulo-corneal and/or irido-corneal adhesions^14^, is the most common cause of congenital corneal opacity^15^. It is thought to arise from defective separation of the lens from the surface ectoderm during early eye development. Peters Plus Syndrome (PPS, MIM261540) combines ASD, primarily Peters anomaly, with short stature and brachydactyly, as well as a variable incidence of cleft palate, intellectual disability, genitourinary anomalies and heart defects^16^,^17^. Classic PPS (obligate triad of Peters anomaly, short stature and brachydactyly) is due to mutations in *B3GLCT*^18^,^19^, previously known as *B3GALTL*, which encodes a β1,3-glucosyltransferase, and is thus a congenital disorder of glycosylation. B3GLCT is an exquisitely specific enzyme, which appends glucose to *O*-linked fucose (*O*-fucose) added by protein *O*-fucosyltransferase 2 (POFUT2) only to thrombospondin type 1 repeats (TSRs). Together, these two enzymes generate a Ser/Thr-linked Glucoseβ1-3Fucose disaccharide^20^. POFUT2 and B3GLCT act in the endoplasmic reticulum and exclusively modify correctly folded TSRs, providing a quality control mechanism regulating the secretion of TSR-containing proteins^21^,^22^,^23^. The specificity of B3GLCT is pre-determined by POFUT2, which modifies Ser/Thr residues (underlined) within the defined consensus sequence CXX(S/T)CXXG in TSRs, which is found in 49 proteins encoded by the human and mouse genomes^24^. Although POFUT2 is related to POFUT1, which adds fucose to epidermal growth-factor-like repeats and is involved in Notch signaling, it has exquisite specificity for TSRs and is not involved in Notch signaling. ASD in PPS likely results from impaired secretion, and thus, a functional loss of one or more TSR-containing proteins indispensable for proper ocular morphogenesis, but which of these proteins are essential in this context is presently undetermined. Furthermore, modification by B3GLCT has only been investigated in a few TSR-containing proteins^21^. Of the 49 TSR-containing proteins having the requisite consensus sequence, ADAMTS (a disintegrin-like and metalloproteinase domain with thrombospondin type 1 motif) proteins are the most numerous, with 26 predicted targets, including 19 secreted proteinases and 7 ADAMTS-like (ADAMTSL) proteins that are not proteinases^25^. ADAMTS proteins have been established to have diverse and crucial roles in development and human disease via analysis of human and animal genetic disorders and engineered mutations^26^.

ADAMTS9 and ADAMTS20 are highly homologous and evolutionarily conserved, reflecting duplication of an ancestral gene^27^. *Adamts9*^*LacZ/LacZ*^ embryos and embryos arising from germline Cre deletion of an *Adamts9* floxed allele (*Adamts9*^*del/del*^) die prior to eye development^28^,^29^. *Adamts20 Belted (bt/bt)* mutant mice are normal but for a white spotting defect and delayed closure of the secondary palate that leads to a low incidence of cleft palate^28^,^30^. *Adamts9* haploinsufficient (*Adamts9*^*del/*+^ or *Adamts9*^*LacZ/*+^) mice survive and are fertile, although they have cardiovascular abnormalities^31^. *Adamts20*^*bt/bt*^; *Adamts9*^*LacZ/*+^ and *Adamts20*^*bt/bt*^; *Adamts9*^*del/*+^ mice die at birth as a result of cleft palate^28^. Here, we demonstrate using *Adamts9*^*del/*+^ mice, that the corneal opacity originally noted postnatally in *Adamts9*^*LacZ/*+^ eyes^32^ is of developmental origin, a result of ASD that includes Peters anomaly and lens abnormalities. Therefore, we investigated *O*-fucosylation of ADAMTS9 to ask whether its deficiency could be a possible mechanism for ASD in PPS patients. We show that ADAMTS9 modification by B3GLCT is required for its secretion, which together with Peters anomaly observed in *Adamts9*^+*/−*^ mice, and its previously defined roles in cardiac and palate development elicited in combination with ADAMTS20, potentially link ADAMTS9 mechanistically to PPS.

## Results

### *Adamts9* haploinsufficiency leads to anterior segment dysgenesis (ASD)

**Figure 1:**
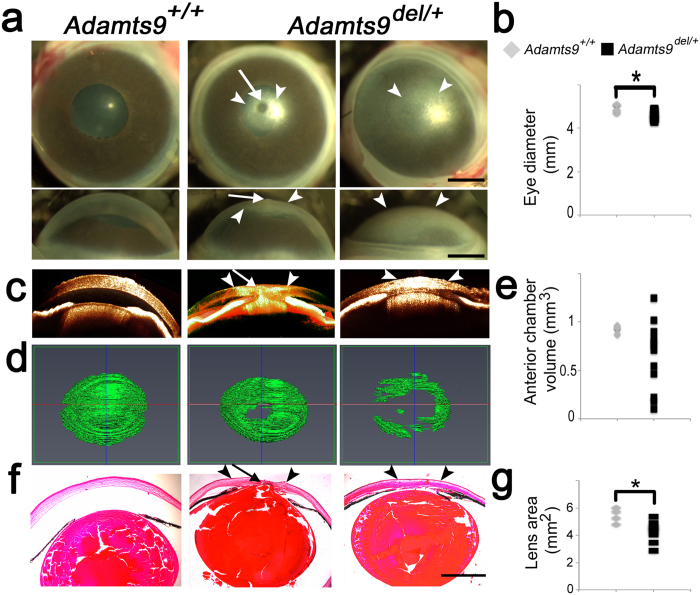
*Adamts9*^*del/*+^ mice have a highly penetrant congenital corneal opacity resulting from ASD. **(a–g)** Corneal opacity in *Adamts9*^*del/*+^ eyes is associated with Peters anomaly (**a**), a smaller eye (**b**), shallow anterior chamber (**c**–**e**), iridocorneal and iridolenticular adhesions (**f**) and a smaller lens (**g**). 3 week-old *Adamts9*^+*/*+^ and *Adamts9*^*del/*+^ enucleated eyes were analyzed by stereomicroscopy (**a**), OCT (**c**,**d**) and H&E staining of paraffin sections (**f**). The arrowheads and arrow indicate, respectively, the corneal opacity and the Peters anomaly. *Adamts9*^*del/*+^ eyes had a significantly smaller diameter than *Adamts9*^+*/*+^ eyes (**b**). Anterior chamber volume was determined from OCT data using the Amira segmentation tool (**d**). Anterior chamber volumes were highly variable in *Adamts9*^*del/*+^ eyes while quite constant in *Adamts9*^+*/*+^ eyes (**e**). Lens area was measured from H&E stained sections and was significantly smaller in *Adamts9*^*del/*+^ eyes as compared to *Adamts9*^+*/*+^ eyes (**g**). The images are representative of 6 *Adamts9*^+*/*+^ and 12 *Adamts9*^*del/*+^ eyes analyzed by OCT. Scale bar = 1 mm. Significance was determined using a 2-tailed student’s *t* test (*p < 0.05).

An *Adamts9* germline mutant (*Adamts9*^*del*^) was generated using a previously described floxed *Adamts9* allele and maintained in the C57BL/6 background^29^. As previously seen in *Adamts9*^*LacZ/*+^ mice^32^, *Adamts9*^*del/*+^ mice had corneal opacities with high penetrance (Fig. 1a and Supplementary Fig. S1a), which were discernible as soon as their eyelids opened (~2 weeks of age). Opacities were bilateral in about half the affected mice, and unilateral in the other half with preferential involvement of the right eye (Supplementary Fig. S1a). The corneal opacities were of variable severity, ranging from faint central clouding to opacity of the entire cornea (Fig. 1a). A central corneal contour anomaly was visible externally in some eyes with corneal opacity (Fig. 1a, central panels). Moreover, *Adamts9*^*del/*+^ eyes were significantly smaller than *Adamts9*^+*/*+^ eyes (Fig. 1b). Optical coherence tomography (OCT) at 3 weeks of age showed a flattened cornea and a shallow anterior chamber in *Adamts9*^*del/*+^ mice compared to the wild-type littermates, comprising little more than a potential space in the most severely affected eyes (Fig. 1c,d). Although the anterior chamber volume was reduced in most *Adamts9*^*del/*+^ eyes, comparison with wild-type chamber volumes did not reach statistical significance owing to the considerable variability of ASD and the presence of buphthalmos (enlarged eye) in at least one *Adamts9*^*del/*+^ eye (Fig. 1e). Peters anomaly, never seen in the wild-type eyes, was identified by OCT in 3-week-old *Adamts9*^*del/*+^ eyes having the central corneal contour anomaly and confirmed histologically by observation of lens-cornea adhesion with disrupted posterior corneal layers, and iridocorneal adhesions (anterior synechiae) (Fig. 1c,f, Table 1). *Adamts9*^*del/*+^ eyes had a smaller lens (Fig. 1g) and some eyes showed ciliary body hypoplasia/dysplasia, signs of vacuolar cataract and persistence of lens fiber nuclei in the posterior lens, none of which were seen in wild-type littermates (Table 1, Supplementary Fig. S1b–d). No histological anomalies of the retina, RPE and choroid were evident in *Adamts9*^*del/*+^ eyes.

### *Adamts9* is expressed at specific sites during mouse eye development

**Figure 2:**
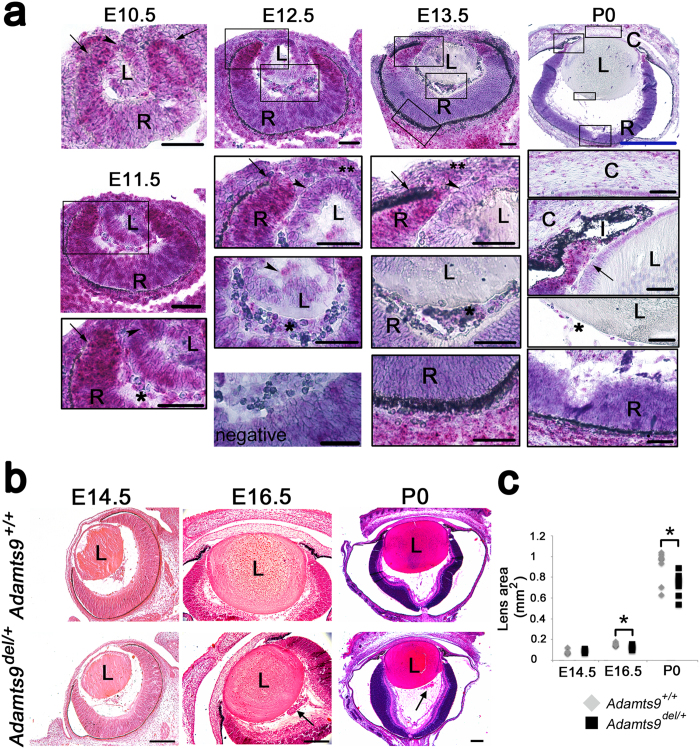
*Adamts9* mRNA is expressed during murine eye development consistent with a developmental sequence of ASD in *Adamts9*^*del/*+^ eyes. (**a**) *In situ* hybridization using an *Adamts9* probe was performed on E10.5 through newborn (P0) *Adamts9*^+*/*+^ eyes. *Adamts9* mRNA expression is visualized as red dots overlying cells, against a purple hematoxylin counterstain. *Adamts9* was expressed throughout this period in the anterior pole of the retina (arrows) and hyaloid vasculature (single asterisk). *Adamts9* expression was also observed in the lens and the lens epithelium (arrowheads) and in mesenchymal cells within the developing cornea (double asterisk). L = lens, R = optic cup/retina, I = iris, C = cornea. The boxed areas in the upper panels are shown at higher magnification (lower black boxed panels). Scale bar = 50 μm; blue scale bar in P0 images = 250 μm. (**b,c**) Sections from *Adamts9*^+*/*+^ and *Adamts9*^*del/*+^ eyes from E14.5 to P0 were stained by H&E and the lens area was measured. At E14.5 *Adamts9*^+*/*+^ and *Adamts9*^*del/*+^ eyes were of comparable size. Posterior to the lens, abnormal material (indicated by an arrow) was observed in the E16.5 and P0 *Adamts9*^*del/*+^ eyes shown here (**b**) but never in *Adamts9*^+*/*+^ eyes. At E16.5 and P0, the *Adamts9*^*del/*+^ lens was significantly smaller than the *Adamts9*^+*/*+^ lens (**c**). Images are representative of at least 3 eyes analyzed at each time point. L = lens. Scale bar = 100 μm. Significance was determined using a 2-tailed Student’s *t* test (*p < 0.05).

To determine the role of *Adamts9* in these anomalies, we defined its spatio-temporal expression pattern in the eye during development (Fig. 2a). At gestational age 10.5 days (E10.5), i.e., shortly after lens vesicle formation, *Adamts9* (red signal) was expressed strongly in the optic cup, preponderantly at its anterior pole, with weak *Adamts9* expression in the invaginating lens vesicle. E11.5 eyes showed the strongest *Adamts9* expression of all developmental stages analyzed, localizing mRNA mostly in the anterior pole of the developing retina/optic cup and more faintly, in the hyaloid vascular plexus and the lens vesicle. At E12.5 and E13.5, strong *Adamts9* expression persisted in the anterior pole of the retina. After E12.5, *Adamts9* mRNA was no longer detected in the lens, but was present in endothelial cells of the tunica vasculosa lentis and vasa hyaloidea propria, and in the choroid and sclera. In newborn eyes, *Adamts9* mRNA was most strongly expressed in the ciliary margin zone and prospective ciliary body, sclera, corneal keratocytes and choroidal vasculature (Fig. 2a). This mRNA distribution pattern was similar to β-galactosidase staining elicited from the intragenic *lacZ* reporter in *Adamts9*^*Lacz/*+^ mice (Supplementary Fig. S2).

### *Adamts9* haploinsufficiency affects lens capsule integrity, its composition, and lens growth

To determine the pathogenic sequence of ASD, and because of continuous expression of *Adamts9* during eye development, *Adamts9*^*del/*+^ and *Adamts9*^+*/*+^ eyes were compared histologically at different developmental stages. H&E staining of E14.5 and older *Adamts9*^*del/*+^ eyes revealed eosinophilic globules adjacent to the posterior aspect of the lens (Fig. 2b, Supplementary Fig. S1e). In contrast, no differences in lens morphology were evident from E10.5–E12.5 (Supplementary Fig. S1f). From E15.5, *Adamts9*^*del/*+^ eyes were smaller than wild-type eyes with a disproportionately smaller lens (Fig. 2b,c and Supplementary Fig. S3a,b).

**Figure 3:**
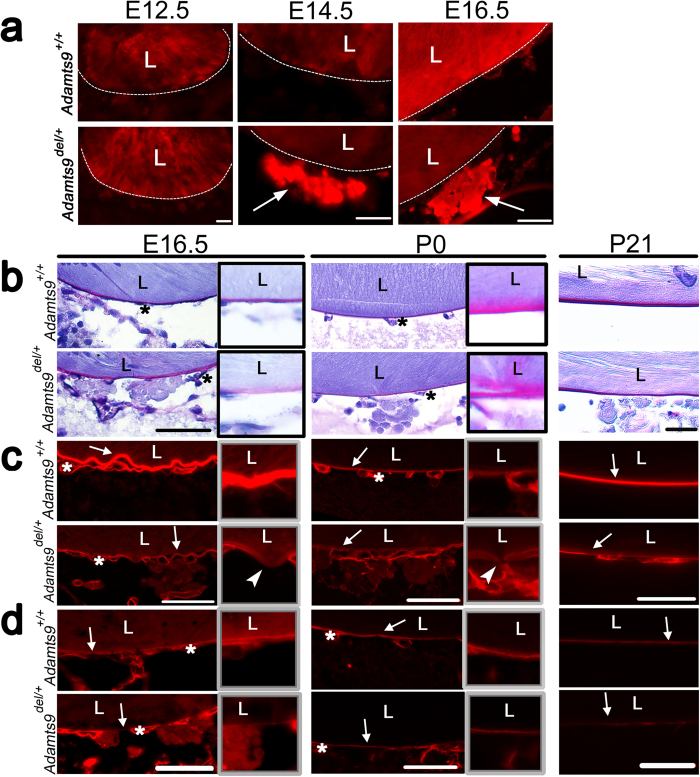
*Adamts9* haploinsufficiency leads to posterior lens extrusions owing to a defective lens capsule. (**a**) E12.5 to E16.5 *Adamts9*^*del/*+^ and *Adamts9*^+*/*+^ eyes were stained using an antibody against γ-crystallin which identified the extrusions lying posterior to the lens as extruded lens fibers (arrows). Images are representative of at least 3 eyes analyzed for each time point. L = lens. Scale bar = 25 μm. (**b–d**) E16.5, P0 and P21 *Adamts9*^*del/*+^ and *Adamts9*^+*/*+^ eyes were stained by the periodic acid Schiff (**b**, PAS stain, magenta, indicative of glycoproteins) or using antibodies directed against collagen IV (**c**, red) or laminin (**d**, red). Collagen IV and laminin immunostaining of the lens capsule (arrow) had reduced intensity in *Adamts9*^*del/*+^ eyes as compared to *Adamts9*^+*/*+^ eyes, most evident in E16.5 embryos. However, the capillary basement membrane of vessels comprising the tunica vasculosa lentis (asterisk) had similar staining in both genotypes. At higher magnification (framed images), segmentally weaker or discontinuous lens capsule immunostaining (arrowheads) suggestive of fenestrae, was observed in mutant eyes. The images are representative of at least 3 eyes analyzed at each time point. L = lens. Scale bar = 50 μm.

The retro-lenticular globules noted from E14.5 were identified as arising from extruded lens fibers using anti-γ-crystallin antibody, suggesting impaired lens capsule integrity (Fig. 3a). Therefore, we characterized the composition and structure of the lens capsule. Periodic acid-Schiff (PAS) staining, an indicator of lens capsule glycoprotein content, was subtly reduced and diffuse in the E16.5 *Adamts9*^*del/*+^ lens capsule (Fig. 3b, insets in left-hand column), although relatively normal in newborn (post-natal (P) 0) and 3-week old (P21) *Adamts9*^*del/*+^ eyes (Fig. 3b, center and right-hand columns). At E16.5, collagen IV immunostaining (Fig. 3c) and laminin immunostaining (Fig. 3d) were conspicuously reduced throughout the circumference of the lens capsule in *Adamts9*^*del/*+^ eyes. At P0, the reduction in collagen IV and laminin immunostaining in the *Adamts9*^*del/*+^ lens capsule was less pronounced than at E16.5 (Fig. 3c,d, center panels). At P21, the lens capsule showed regions where collagen IV and laminin immunostaining of the lens capsule were interrupted (Fig. 3c,d, right-hand panels). In contrast, collagen IV and laminin staining in capillaries comprising the tunica vasculosa lentis, the vascular network adjacent to the embryonic lens, were not altered in *Adamts9*^*del/*+^ eyes (Fig. 3c,d).

Versican, fibrillin-2 and fibronectin, which are major components of embryonic ECM, were analyzed by immunofluorescence (Supplementary Fig. S4). Although versican is a known ADAMTS9 substrate, no difference in staining intensity or distribution of versican was detected between *Adamts9*^*del/*+^ and *Adamts9*^+*/*+^ eyes at E12.5 and E14.5 (Supplementary Fig. S4). Both fibrillin-2 and fibronectin were present around the lens at E12.5, especially, in the hyaloid tissue (Supplementary Fig. S4). Fibrillin-2 immunostaining was more intense at E12.5 and E14.5 in *Adamts9*^*del/*+^ eyes while fibronectin immunostaining was increased in *Adamts9*^*del/*+^ eyes at E14.5, but not at E12.5 (Supplementary Fig. S4). In contrast, a consistent difference in fibrillin-2 or fibronectin immunostaining between *Adamts9*^*del/*+^ and *Adamts9*^+*/*+^ eyes older than E14.5 was not observed, indicating a transient alteration of hyaloid ECM from E12.5 to E14.5 (Supplementary Fig. S4).

**Figure 4:**
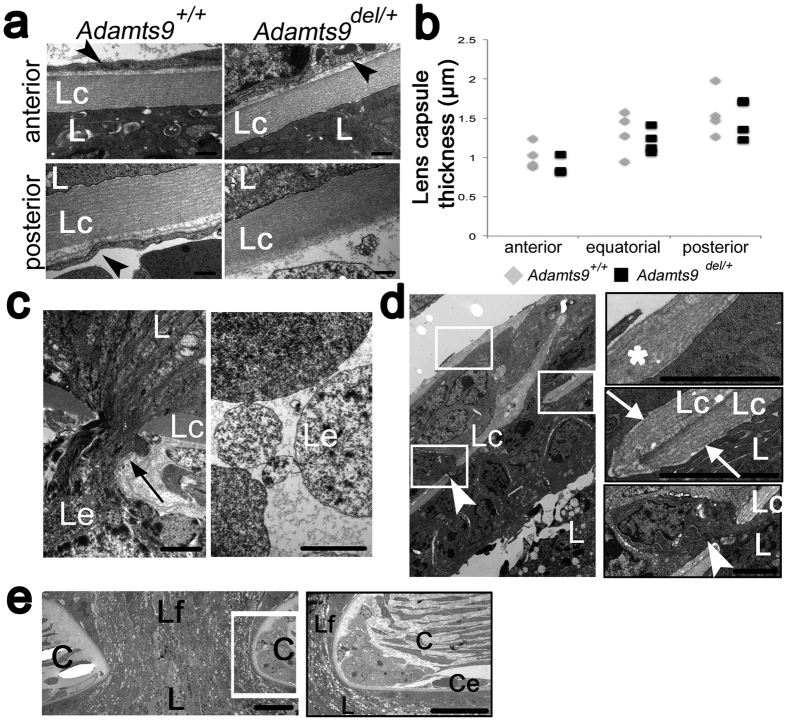
Transmission electron microscopy (TEM) identifies lens capsule fenestrae in E16.5 *Adamts9*^*del/*+^ eyes. (**a**) The anterior and posterior lens capsule were of comparable appearance in *Adamts9*^*del/*+^ and *Adamts9*^+*/*+^ eyes through most of their extent. Arrowheads indicate endothelial cells of microvessels in the pupillary membrane (anterior) or the tunica vasculosa lentis (posterior). Panels are representative of 4 eyes of each genotype. Scale bar = 500 nm. (**b**) Anterior, equatorial and posterior lens capsule thickness was similar in *Adamts9*^*del/*+^ and *Adamts9*^+*/*+^eyes. (**c**) A defect in the posterior lens capsule associated with a lens fiber extrusion in an *Adamts9*^*del/*+^ eye (left-hand panel). The right-hand panel shows that the extruded material lying in the vitreous is membrane-bound. Scale bar = 2 μm. (**d)** In *Adamts9*^*del/*+^ eyes, lens epithelial cells traversed the discontinuous anterior lens capsule (white arrowhead) and formed nests of cells surrounded by basement membrane similar to the lens capsule. Higher magnifications of the indicated regions are shown as framed panels on the right. The asterisk in the upper panel shows the extraneous anterior layer of lens capsule formed by the ectopic cells, the center panel shows a local duplication or folding of the lens capsule, and the lower panel shows a lens epithelial cell (arrowhead) penetrating the fenestrated lens capsule. Scale bar = 2 μm. (**e**) In an *Adamts9*^*del/*+^ eye, Peters anomaly was identified by continuity of the lens with surface ectoderm across a discontinuous cornea (left-hand panel). The right-hand panel shows the resulting corneal edge at high magnification to indicate the interrupted corneal endothelium. Scale bar = 2 μm. L = lens, C = cornea, Lc = lens capsule, Le = lens extrusion, Lf = lens fibers, Ce = corneal endothelium. Significance was determined using a 2-tailed student’s *t* test.

Despite the marked differences in lens capsule immunostaining between *Adamts9*^*del/*+^ and *Adamts9*^+*/*+^ eyes at E16.5, transmission electron microscopy (TEM) at this age showed comparable lens capsule appearance, including thickness, layering and electron density over almost the entire extent of the lens capsule in *Adamts9*^*del/*+^ and *Adamts9*^+*/*+^ eyes (Fig. 4a,b). Notwithstanding this apparent normalcy, discontinuities, i.e., fenestrations in the lens capsule were also detected by TEM in *Adamts9*^*del/*+^ eyes (Fig. 4c,d). Posteriorly, *Adamts9*^*del/*+^ eyes contained extruded material continuous with lens fibers (Fig. 4c) and anteriorly, TEM revealed that lens epithelium cells had translocated through lens capsule fenestrae into the anterior chamber (Fig. 4d). The translocated cells were surrounded by an ECM with a similar ultrastructural appearance as the lens capsule, and a duplicated lens capsule was observed anterior to the ectopic cell nests (Fig. 4d). In one eye analyzed by TEM, we observed continuity of the lens fibers with corneal stroma, along with an interrupted corneal endothelium and Descemet’s membrane, together satisfying identification of Peters anomaly (Fig. 4e).

### *Adamts9* ^*del/*+^ lens epithelium undergoes epithelium to mesenchyme transition

**Figure 5:**
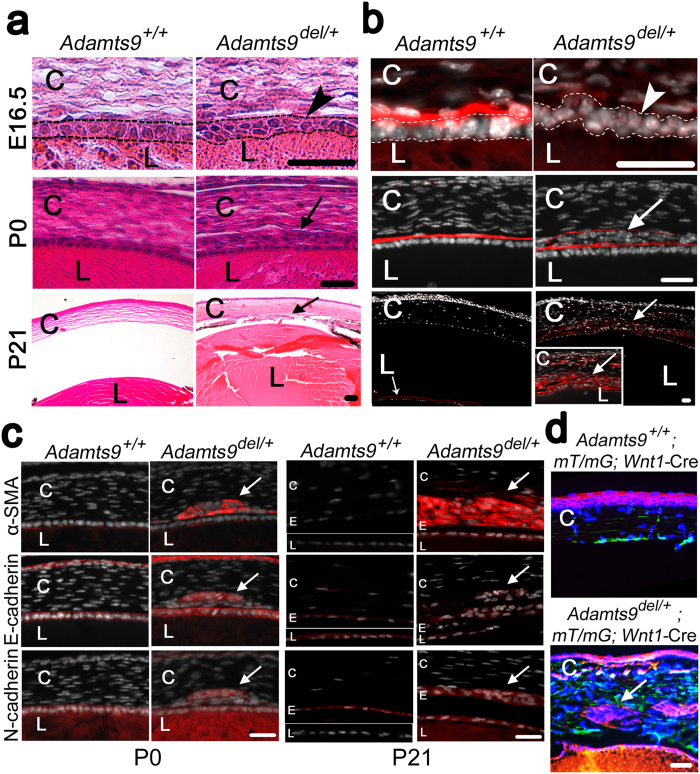
*Adamts9*^*del/*+^ eyes have aberrant and intra-corneal nests of cells arising from epithelium-mesenchyme transformation of lens epithelium. (**a,b**) E16.5, P0 and P21 *Adamts9*^+*/*+^ and *Adamts9*^*del/*+^ eyes were stained with H&E (**a**) or an anti-collagen IV antibody (red) and the nuclei were counterstained with DAPI (white) (**b**). At E16.5, a disorganized lens epithelium (arrowhead) was associated with weaker collagen IV staining in *Adamts9*^*del/*+^ eyes. At P0 and P21, aberrant nests of cells surrounded by collagen IV-positive ECM were observed only in the *Adamts9*^*del/*+^ eyes (arrow). Higher magnification of these cells in P21 *Adamts9*^*del/*+^ eyes is shown (inset). These images represent at least 3 eyes analyzed at each time point. L = lens, C = cornea. Scale bar = 25 μm. (**c**) P0 and P21 *Adamts9*^*del/*+^ and *Adamts9*^+*/*+^ eyes were stained using antibodies directed against α-smooth muscle actin (α-SMA) (red), E-cadherin (red) or N-cadherin (red) and nuclei were stained by DAPI (nucleus: white). At P0 and P21, the ectopic cells in *Adamts9*^*del/*+^ corneas stained positive for α-SMA, E-cadherin and N-cadherin (arrows) whereas no α-SMA expression was observed in the lens or the cornea of *Adamts9*^+*/*+^ mice, and E-cadherin was distinctly expressed by the lens and corneal epithelium. The corneal endothelium of *Adamts9*^+*/*+^ mice strongly expressed N-cadherin, but also low levels of E-cadherin. C = cornea, E = corneal endothelium, L = lens. (**d**) *Adamts9*^*del/*+^ and *Adamts9*^+*/*+^ mice were bred with *mT/mG; Wnt1*-Cre mice for lineage tracing of neural crest-derived cells (green). In adult *Adamts9*^*del/*+^*; mT/mG; Wnt1-Cre* eyes (lower panel), ectopic cells are red (arrow) and thus, unlike corneal keratocytes and corneal endothelium (green, see *mT/mG; Wnt1-Cre* cornea in upper panel), they do not arise from the neural crest. Scale bar = 25 μm.

The lens epithelium normally comprises a single layer of cells arranged in a columnar format reflecting strict apical-basal polarity, as was seen in *Adamts9*^+*/*+^ embryos (Fig. 5a,b). In contrast, up to 3 layers of lens epithelial cells were present in E16.5 and P0 *Adamts9*^*del/*+^ eyes (Fig. 5a,b). In P21 *Adamts9*^*del/*+^ eyes, lens epithelium cells were embedded in the corneal stroma (Fig. 5a,b). Combined with staining for laminin, DAPI stained nuclei indicated that the cells adopted aberrant orientations in mutant E16.5 eyes, indicating disruption of cell polarity (Fig. 5b, upper panel). At birth, we observed aberrant nests of cells anterior to the lens capsule and within the cornea that were morphologically distinct from corneal keratocytes, which have a flattened nucleus and an abundant cytoplasm (Fig. 5b, center panels). ECM surrounding these ectopic cells stained positive for laminin and collagen IV, indicative of ectopic basement membrane (Fig. 5b, center panel), consistent with the TEM observations of a multilayered lens epithelium and duplicated anterior lens capsule (Fig. 4d). At 21 days of age, the cell nests were more prominent than at birth, and were embedded in an abundant collagen IV and laminin (Fig. 5b, lower panel, inset). We concluded that the aberrant cell nests in the anterior chamber and corneal stroma were ectopic lens epithelium cells that continued to deposit lens capsule after E16.5. In addition, the keratocyte density was greater in the P21 *Adamts9*^*del/*+^ corneal stroma than the *Adamts9*^+*/*+^ littermates (Supplementary Fig. S5). However, cell density in the corneal stroma and corneal thickness were not significantly altered in embryonic or newborn *Adamts9*^*del/*+^ eyes as compared to *Adamts9*^+*/*+^ eyes (Supplementary Fig. S5), indicative of post-natal corneal changes.

Corneal invasion by lens epithelium suggested occurrence of epithelium to mesenchyme transition (EMT) of lens epithelium. During EMT, epithelial cells lose or reduce expression of epithelial markers, such as E-cadherin, and acquire mesenchymal markers, such as N-cadherin and α-smooth muscle actin (α-SMA), as well as altered polarity. In *Adamts9*^*del/*+^ newborn eyes, the anteriorly extruded lens epithelium cells expressed the mesenchymal markers α-SMA and N-cadherin, as well as the epithelial marker, E-cadherin (Fig. 5c). In *Adamts9*^+*/*+^ eyes, α-SMA staining was absent in corneal stroma and lens epithelium, whereas E-cadherin was expressed only by the lens and the corneal epithelium, and N-cadherin by the lens epithelium and lens fibers (Fig. 5c). At P21, ectopic lens epithelium in *Adamts9*^*del/*+^ eyes still expressed the three markers, although α-SMA immunostaining was stronger than at P0, and E-cadherin expression was reduced (Fig. 5c). In P21 *Adamts9*^+*/*+^ eyes, α-SMA staining was not detectable in the cornea or the lens, and E-cadherin and N-cadherin were only expressed, as expected, by lens epithelium and corneal endothelium, respectively. These changes are indicative of age-related progression of EMT of *Adamts9*^*del/*+^ ectopic lens epithelial cells, while retaining some characteristics of the lens epithelium. Since lens epithelium does not express *Adamts9* (Fig. 2a, Supplementary Fig. S2), EMT may be secondary to lens capsule alteration and loss of appropriate lens epithelium basal contacts with the lens capsule.

To further establish the origin of the ectopic cell nests, *Adamts9*^*del/*+^ and *Adamts9*^+*/*+^ mice were crossed with *Wnt1*-Cre mice carrying a dual fluorescent reporter (*mT/mG)*. In *Adamts9*^+*/*+^*; mT/mG; Wnt1*-Cre eyes, neural crest-derived cells (e.g., keratocytes in the cornea and corneal endothelium) were marked by green fluorescence, reflecting excision of the Td Tomato reporter (red) by Cre and a switch to GFP expression, whereas corneal epithelium (Fig. 5d, top panel) and lens epithelium displayed constitutive red fluorescence. In *Adamts9*^*del/*+^*; mT/mG; Wnt1*-Cre eyes, the aberrant cell nests in the anterior chamber exhibited red fluorescence, demonstrating that in contrast to corneal stroma (green), these cells were not neural crest-derived (Fig. 5d, lower panel). Taken together with the presence of lens capsule components detected by immunostaining around these cells, and lens epithelium-like morphology in TEM (Fig. 4) we suggest that the ectopic cells in mutant cornea arise from lens epithelium by EMT.

### POFUT2 and B3GLCT modify ADAMTS9 to regulate its secretion

**Figure 6:**
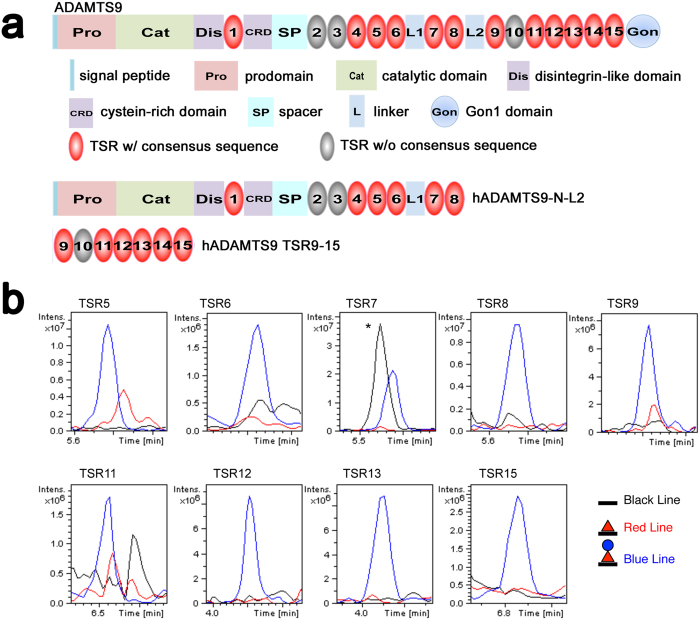
Specific ADAMTS9 TSRs are *O*-fucosylated. (**a**) Domain structure of ADAMTS9 and of the ADAMTS9 constructs used for mass spectrometry (hADAMTS9-N-L2 and hADAMTS9 TSR9-15). TSRs that carry a consensus sequence for *O-*fucosylation are shown in red. (**b**) Extracted ion chromatograms showing the relative amounts of the unmodified form (black line), *O-*fucose monosaccharide form (red line, fucose indicated by red triangle), and Glucoseβ1-3Fucose disaccharide form (blue line, glucose indicated by blue circle) of peptides from TSRs 5–9, TSRs11-13 and TSR15 of ADAMTS9 generated using the corresponding masses listed in Appendix Table S1. Note that lack of data for TSRs 1, 4, and 14 does not indicate that they are unmodified, but that the ions corresponding to those sites were not detected. (*=contaminating peptide).

**Figure 7:**
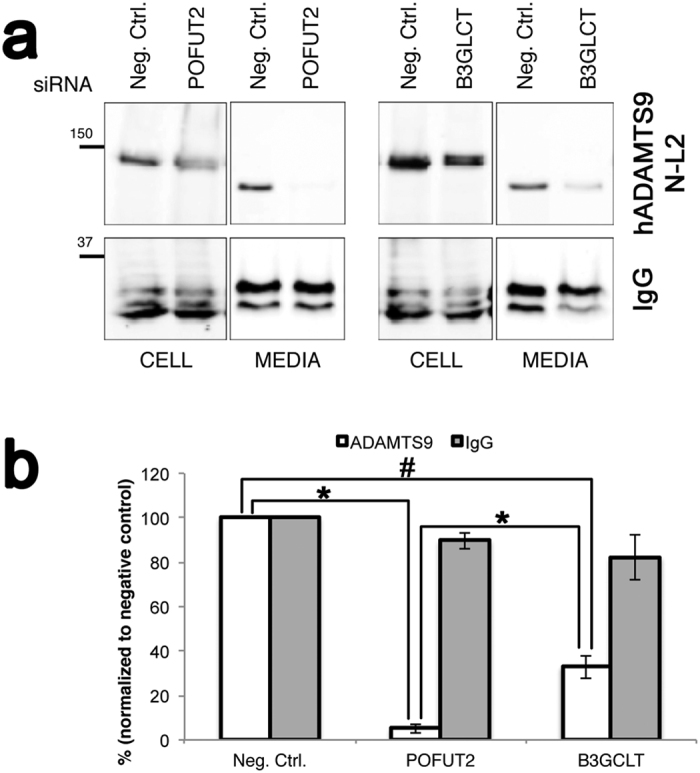
POFUT2 is indispensable for ADAMTS9 secretion whereas B3GLCT is required for optimal secretion. (**a**) HEK293T cells were co-transfected with siRNA targeting *POFUT2,* or *B3GLCT*, or a non-targeting control (Neg. Ctrl.), and hADAMTS9-N-L2 or human IgG heavy chain. Media and lysates were analyzed by western blotting with anti-myc (hADAMTS9-N-L2) or anti-human IgG. ADAMTS-N-L2 in medium migrates more rapidly compared to the cell lysate because the N-terminal propeptide (~25 kDa) is excised by furin at the cell-surface. The western blot images are cropped from gels which were provided for review as Supplementary information (Supplementary Fig. S7). (**b**) Quantitation of data from the western blot is shown relative to the control siRNA (Neg. Ctrl.). Data are mean^+/−^ standard deviation (n = 3). Significance was determined using a 2-tailed Student’s *t* test (*p < 0.005, ^#^p < 0.001).

ADAMTS9 contains 15 TSRs, of which 12 contain the *O*-fucosylation consensus motif, CXX(S/T)CXXG (Fig. 6a). Full-length ADAMTS9 is secreted into the medium of transfected cells at levels too low to permit efficient recombinant protein purification, and natural sources of this molecule are unavailable. Therefore, to determine whether ADAMTS9 TSRs are modified by POFUT2 and B3GLCT, we generated two recombinant human ADAMTS9 constructs (Fig. 6a), that, between them, include all its TSRs, for analysis by mass spectral glycoproteomic methods^22^,^23^. These constructs, containing the first 8 TSRs (hADAMTS9-N-L2) or TSR9-15 (hADAMTS9 TSR9-15) (Fig. 6a), were purified from the conditioned medium of stably transfected HEK cells and were subjected to tryptic digestion. Mass spectral analysis of the peptides confirmed modification of TSR5-9, TSR11-13 and TSR15 by POFUT2 and B3GLCT (Fig. 6b, Supplementary Table S1 and Supplementary Fig. S6). Semi-quantitative analysis by extracted ion chromatography (EIC) showed the fully modified, Glucoseβ1-3Fucose disaccharide forms of the peptides to be the most abundant (Fig. 6b). Since POFUT2 and B3GLCT are the only known enzymes capable of adding these two sugars to TSRs^33^,^34^,^35^,^36^,^37^, these data strongly support the conclusion that ADAMTS9 is modified by both POFUT2 and B3GLCT. Whether or not *O*-fucosylation governed quality control of ADAMTS9 for secretion was determined by knockdown of *POFUT2* or *B3GLCT* mRNA using specific siRNAs and a scrambled siRNA as a control in HEK cells. The secreted ADAMTS9-N-L2 levels in the medium of *POFUT2, B3GLCT* or control siRNA transfected cells were compared relative to the impact on secretion of a co-transfected plasmid expressing IgG (Fig. 7a, complete gel is shown in Supplementary Fig. S7). As previously seen, ADAMTS9-N-L2 migrated more rapidly in the medium of transfected cells than in the cell lysate (Fig. 7a, Supplementary Fig. S7) because of furin-mediated excision of the N-terminal propeptide (Fig. 7a) which reduces molecular mass by ∼25kDa^38^. Knockdown of *POFUT2* led to a nearly complete loss of ADAMTS9-N-L2 secretion compared to the scrambled siRNA (Fig. 7a,b, Supplementary Fig. S7). Knockdown of *B3GLCT* also caused a statistically significant reduction of ADAMTS9 secretion (Fig. 7b). In contrast, knockdown of *POFUT2* or *B3GLCT* had no effect on secretion of transfected IgG chain, which contains no TSRs (Fig. 7a,b, Supplementary Fig. S7). Thus, modification of ADAMTS9 with the Glucoseβ1-3Fucose disaccharide was required for its optimal secretion. Although ADAMTS9 carrying only *O*-fucose was also secreted after *B3GLCT* knockdown, it was at much lower levels than in the control cells (Fig. 7a,b).

Although the present studies clearly demonstrated ADAMTS9 post-translational modification by POFUT2 and B3GLCT, and B3GLCT has long been known as the causative gene in PPS, there is little known about the spatial and temporal regulation of expression of these transferases during eye developement. Because this information is crucial for providing a physiological context for the action of these enzymes on ADAMTS9, we determined their mRNA expression pattern using ISH. As shown in Supplementary Fig. S8, both *Pofut2* and *B3glct* were expressed in the developing eye from E11.5 to birth. Their mRNAs were widely expressed, being evident in the optic cup/retina, lens epithelium, cornea and peri-ocular mesenchyme. They not only overlap entirely with *Adamts9* mRNA distribution (Fig. 2a, Supplementary Fig. S2), but show a broader expression than *Adamts9* mRNA consistent with their having 48 other potential targets, which may each have very different expression patterns. We conclude that *Pofut2* and *B3glct* mRNAs are expressed in the very same cells that express *Adamts9* and are therefore, physiologically relevant to ADAMTS9 post-translational modification and secretion in the eye.

## Discussion

We demonstrate here that congenital corneal opacity and Peters anomaly results from *Adamts9* haploinsufficiency in mice. Because enzyme deficiencies are typically recessive in their manifestation, the hemizygous impact of *Adamts9* is remarkable and implies a major role for this gene in ocular morphogenesis. Corneal opacity occurs in two distinct *Adamts9* alleles, *Adamts9*^*del/*+^ and *Adamts9*^*LacZ/*+^, but not in mice hemizigous for a hypomorphic gene trap allele *Adamts9*^*GT*^, which has ADAMTS9 function intermediate between the wild-type and haploinsufficient^39^. Together, these observations indicate that a critical dosage threshold of ADAMTS9 is involved in eye development. *Adamts9*^*LacZ/*+^ and *Adamts9*^*del/*+^ alleles differed in the timing of detection of the ocular defect. In *Adamts9*^*LacZ/*+^ mice, corneal opacity was evident in only 20% after 5 weeks of age and in 80% after 25 weeks of age^32^, whereas in *Adamts9*^*del/*+^ mice, the corneal opacity was obvious as early as 2 weeks of age and present in nearly all mice by 3 weeks. Although Peters anomaly was not observed in *Adamts9*^*LacZ/*+^ mice, anterior synechiae (lens-iris-cornea adhesions) and lens extrusions similar to those detected in *Adamts9*^*del/*+^ mice were present. These differences could result from different targeting strategies employed in the two alleles or slightly different genetic backgrounds of the two strains. The *Adamts9*^*LacZ/*+^ allele was generated in a hybrid genetic background (129Sv X C57Bl/6) by targeting exon 3 (encoding the propeptide), and was backcrossed for 10 generations into the C57Bl/6 background^32^, whereas the *Adamts9*^*del/*+^ allele was generated and subsequently maintained in the C57Bl/6 background, and it was engineered to lack exons 5-8 (encoding the catalytic domain)^29^. Each targeted allele results in a frame-shift and the *Adamts9* mRNA, if stable, would generate only the N-terminal propeptide in the mutants, to which no innate activity has been ascribed in any ADAMTS protease. Consistent with a possible role for the genetic background, the ocular phenotype was not initially apparent in 129Sv X C57Bl/6 *Adamts9*^*LacZ/*+^ mice, and was only noted after six to eight crosses into the C57Bl/6 strain^32^. Although it was previously reported that eye abnormalities occur spontaneously at low frequency in C57Bl/6 mice, and have a strong predilection for the right side^40^,^41^, we did not notice eye anomalies in wild type littermates and the preponderance of anomalies in the right eye of *Adamts9*^*del/*+^ mice is presently unexplained.

Although *Adamts9*^*del/*+^ eyes do not exhibit morphologic anomalies prior to E12.5, the presence of Peters anomaly indicates that lens separation, which occurs at E11, is compromised in a significant proportion of eyes. After the lens separation period, *Adamts9*^*del/*+^ eyes consistently showed impaired lens growth and loss of structural integrity of the lens capsule resulting in lens fiber extrusion posteriorly, and migration of the lens epithelium into the cornea anteriorly through an EMT-like process. The lens capsule is a specialized and exceptionally thick basement membrane composed mainly of collagen IV, laminin, entactin/nidogen and perlecan^42^. It is deposited by lens epithelium anteriorly and lens fibers posteriorly as successive basement membrane layers during development. PAS stain and immunostaining demonstrated that the lens capsule composition was transiently altered during development of *Adamst9*^*del/*+^ eyes. Other mouse mutants reported to have lens extrusions and/or outgrowth of lens epithelium had defined defects in the lens capsule components, i.e., perlecan^43^ and laminin^44^. *Col4a1* mutant mice develop ASD, although lens fiber extrusions through the lens capsule were not described^13^,^45^. We conclude that ADAMTS9 may participate in stabilization of basement membrane or in lens capsule remodeling during lens growth. Such a role, if compromised, could also have an adverse effect on “pinching off” of the lens and result in Peters anomaly. Interestingly, mice deficient in peroxidasin, an enzyme responsible for collagen IV crosslinking through formation of sulfilimine bonds^46^, developed ASD associated with a small lens, loss of lens capsule integrity and posterior and anterior lens extrusions^47^. These mice also have white spotting closely resembling the pigmentation defect in *Adamts20*^*bt/bt*^ mice^48^ which is exacerbated in *Adamts20*^*bt/bt*^*;Adamts9*^*del/*+^ newborns^28^. We speculate that these findings could suggest that ADAMTS9 and peroxidasin may work in the same developmental pathway, a potential future direction for this work. *Adamts9* haploinsufficiency may affect assembly of collagen IV or laminin subunits expressed in the embryonic period, such as laminin α1, which is essential for embryonic lens capsule development, and collagen IV assemblies with chain composition α1α1α2:α1α1α2 and α1α1α2:α5α5α6, which are expressed in the embryonic lens capsule and replaced at birth by collagen IV with the chain composition α3α4α5:α3α4α5^42^. The ADAMTS9 substrate versican is present in the hyaloid space from E10-E14, but its staining in the mutant eyes was not consistently different from the wild-type.

A proper interaction of the lens epithelial cells with the lens capsule at their basal aspect maintains their polarity, as demonstrated in integrin and integrin-linked kinase knockout mice^49^,^50^,^51^. Our findings indicate that lens epithelium cells transitioned to an intermediate EMT state between P0 and P21, since they deposited lens capsule-like basement membrane, identified by immunostaining and TEM analysis and retained E-cadherin expression, yet lost their polarity and acquired α-SMA and N-cadherin expression. In addition to Peters anomaly, corneal invasion by lens epithelium, lens capsule deposition in the corneal stroma, as well as increased keratocyte density are together likely to be significant contributors to corneal opacity. We propose that the consecutive changes we observed comprise a pathogenic sequence arising from a flawed lens capsule.

Of 49 known B3GLCT targets, ADAMTS9 is the first to be associated with Peters anomaly. The similarities between the ocular phenotype of *Adamts9*^*del/*+^ mice and the ocular phenotype of PPS in humans suggests ADAMTS9 as the first B3GLCT substrate whose impairment potentially explains the ocular anomalies of PPS. In strong support of this possibility, we have further shown, **1**. That several ADAMTS9 TSRs are *O*-fucosylated, **2**. That *B3GLCT* or *POFUT2* knockdown, i.e. defective *O*-fucosylation, is essential for ADAMTS9 secretion and **3**. That *Adamts9, B3glct* and *Pofut2* have overlapping expression in the eye throughout development. POFUT2 and B3GLCT likely affect ADAMTS9 secretion by a quality control process occurring in the ER that was previously established by analysis of ADAMTSL1, ADAMTSL2 and ADAMTS13, which showed that the modifying enzymes recognized properly folded TSRs and stabilized them by glycosylation^21^. Because TSR modification by B3GLCT is dependent on prior attachment of *O*-linked fucose, POFUT2 knockdown inevitably affects both monosaccharide and disaccharide forms of *O*-fucosylation. Consistent with the severe reduction of ADAMTS9 secretion upon POFUT2 knockdown, *Pofut2* null embryos, like *Adamts9* nulls embryos, die in early development with essentially identical phenotypes^37^,^52^, although *Pofut2*^+*/−*^ mice do not have eye anomalies. In contrast, knockdown of B3GLCT reduced, but did not eliminate ADAMTS9 secretion *in vitro*, predicting a milder outcome than loss of POFUT2. Although *B3glct* deficient mice are currently unavailable for comparison with *Pofut2* and *Adamts9*-deficient mice, survival of humans with PPS supports the milder-than-expected outcome of B3GLCT than POFUT2 deficiency. ADAMTS9, in addition to the ASD reported here, has additional relevance to PPS. Notably, *Adamts9* haploinsufficiency resulted in cardiovascular defects and, in combination with *Adamts20* deficiency, cleft palate, which are anomalies seen frequently in PPS^28^.

Since *Adamts9*^*del/*+^ mice were of normal size and limb-specific *Adamts9* conditional deletion did not affect limb length^29^, impairment of ADAMTS9 does not explain short stature in PPS. PPS is predicted to be a composite phenotype resulting from reduced secretion (and therefore functional loss) of a subset of developmentally crucial B3GLCT substrates from among 49 predicted targets. Of these, not all are likely to be subjected to quality control by B3GLCT, so the number of functionally impaired targets in PPS is likely to be even smaller. Which are some of the other likely participants in PPS from among the 26 target ADAMTS proteins? Geleophysic dysplasia, which is caused by recessive *ADAMTSL2* mutations, results in short stature, and brachydactyly, which are two major defining characteristics of PPS, and affected individuals have similar facial features to PPS^17^,^53^. Appropriately, *B3GLCT* knockdown abolished ADAMTSL2 secretion^21^. *ADAMTS10* mutations lead to short stature and brachydactyly in Weill-Marchesani syndrome^54^, whereas *ADAMTS17* mutations lead to short stature^55^. Thus, ADAMTS10 and ADAMTS17 are potentially relevant to PPS, but the effect of B3GLCT on their secretion is presently unknown. On the other hand, some potential targets could be excluded. ADAMTS2 and ADAMTS13 are required for skin integrity and hemostasis respectively, but neither function is impaired in PPS, and, indeed, ADAMTS13 is not subject to B3GLCT quality control^21^. From these observations, it seems reasonable to conclude that impairment of ADAMTS9 (in the eye, palate and heart) and ADAMTSL2 (in skeletal growth) contributes to the PPS phenotype, but that other relevant targets remain to be evaluated.

Intriguingly, both *ADAMTS9* and *B3GLCT* were recently identified in genome-wide association studies as loci linked to age-related macular degeneration^56^,^57^,^58^. The direct functional link between their protein products demonstrated here for the first time, expression of *Adamts9* in the microvasculature^32^, and its role in eye development strengthens this bi-allelic association. Future functional analysis of *ADAMTS9* and *B3GLCT* in age-related macular degeneration is thus warranted. In addition, the mouse eye phenotype reported here suggests consideration of *ADAMTS9* itself as a candidate gene for ASD. Although *Adamts9* mRNA was strongly expressed in the anterior pole of the optic cup, especially in E11.5 eyes, the current study demonstrated severe ASD, and not retinal anomalies. Because the eye develops from crosstalk between the optic cup and lens ectoderm, this suggest that ADAMTS9, a cell-surface and secreted protease produced mainly by the optic cup and hyaloid plexus, may well act in *trans* on the lens vesicle. Future experiments using *Adamts9* conditional mutagenesis in specific ocular structures or embryonic lineages contributing to the eye will be insightful in this regard.

## Materials and Methods

### Mouse alleles and genotyping

Mice with targeted germline inactivation of *Adamts9* (referred to as *Adamts9*^*del/*+^) using the *Adamts9* floxed allele (RRID: JAX_026103) and their genotyping were described previously^29^. *Gt(ROSA)26Sor*^*tm4(ACTB-tdTomato,-EGFP)Luo*^ mice^59^ (RRID: MGI_3722405), referred to as *mT/mG*, were from Jackson Laboratories (Bar Harbor, ME). Detection of the *mT/mG* transgene was done using PCR primers recommended by Jackson Laboratories (http://jaxmice.jax.org/). *Wnt1-cre* mice (B6.Cg-Tg(Wnt1-cre)11Rth Tg(Wnt1-GAL4)11Rth/J; Jackson Laboratories, Bar Harbor, ME; RRID: IMSR_JAX:009107) were bred with *mT/mG* mice and *Adamts9*^*del/*+^ mice to obtain *Adamts9*^*del/*+^ and *Adamts9*^+*/*+^ mice with conditional deletion of the membrane-bound TdTomato reporter and expression of GFP in neural crest cell-derived tissues. *Wnt1*-Cre mice were genotyped using the following primers: *Wnt1* forward = 5′-TAAGAGGCCTATAAGAGGCGG-3′; *Wnt1* reverse = 5′-CGCATAACCAGTGAAACAGCATTGC-3′. For timed pregnancies, the date of the vaginal plug was designated E (embryonic age) 0.5. All analyses of *Adamts9*^*del/*+^ eyes used wild-type littermates as controls.

### Gross Morphology and Optical Coherence Tomography

We evaluated adult mouse eyes for the presence of leukoma (corneal opacity) using a Leica MZ6 stereomicroscope coupled to an Insight Spot2 FireWire camera (Diagnostic Instruments, Inc, Sterling Heights, MI) immediately after dissection and immersion in PBS. E12.5 and E15.5 mouse embryos were fixed in 4% paraformaldehyde (PFA) and their eyes were photographed. The eye surface and eye diameter was measured using ImageJ^®^ software (NIH, Bethesda, MD). At E15.5, the lens surface area, rendered opaque by 4% PFA fixation, was also measured using ImageJ^®^ software.

Optical Coherence Tomography (OCT) was performed on enucleated P21 mouse eyes fixed overnight in 4% PFA. The eyes were imaged using a custom-built Fourier domain OCT system with a quasi-telecentric scanner, linear-in-wavenumber spectrometer and a line-scan camera with a line rate of 47 kHz^40^,^41^. The axial resolution as well as the lateral resolution is approximately 10 μm in tissue. Custom MATLAB programs (MathWorks; Natick, MA) were utilized to create OCT images from the raw data. Amira software (FEI Visualization Sciences Group; Burlingtong, MA) was used to visualize OCT data and quantify anterior chamber volume. All mouse work was performed under a protocol approved by the Cleveland Clinic Institutional Animal Care and Use Committee. Animal husbandry and euthanasia was done in accordance with guidelines established by the American Veterinary Medical Association.

### Histology, immunohistochemistry (IHC) and electron microscopy

Mouse embryos or enucleated eyes were fixed overnight at 4 °C in 4% PFA prior to paraffin embedding, and 5 μm thick sections were taken for hematoxylin and eosin (H&E) stain, periodic acid-Schiff stain, oxytalan fiber staining, or immunohistochemistry/immunofluorescence. Cornea cell density and thickness were quantified on DAPI stained sections using ImageJ^®^ software.

For immunofluorescence, sections were subjected to microwave heating in 10 mM sodium citrate-EDTA pH 6.2 for 4 × 1.5 min, followed by gradual cooling to ambient temperature or by incubation with 5 μg/ml proteinase K (Invitrogen, Carlsbad, CA) for 3 min at room temperature (for anti-laminin and anti-collagen IV immunostaining). The sections were then incubated with the following antibodies: anti-γ-crystallin rabbit polyclonal antibody (dilution:1/200; catalog number FL-175, sc-227346, Santa Cruz Biotechnology, Santa Cruz, CA), anti-collagen IV rabbit polyclonal antibody (dilution 1/500; catalog number 600-401-106S, Rockland, Gilbertsville, PA), anti-laminin rabbit polyclonal antibody (dilution: 1/400; catalog number L9393, Sigma-Aldrich, St-Louis, MO), anti-N-cadherin mouse monoclonal antibody (dilution:1/100; catalog number 610920, BD Transduction Laboratories, San Jose, CA), anti-E-cadherin mouse monoclonal antibody (dilution:1/100; catalog number 610181, BD Transduction Laboratories), anti-α-smooth muscle actin Cy3 coupled mouse monoclonal antibody (dilution:1/200; catalog number C6198, Sigma-Aldrich), anti-fibrillin-2 rabbit polyclonal antibody (dilution:1/500; kindly provided by Robert Mecham^60^), anti-fibronectin rabbit polyclonal antibody (dilution:1/500; catalog number AB2033, Millipore, Billerica, MA), anti-versican GAGβ rabbit polyclonal antibody (dilution: 1/500, catalog number AB1033, Millipore), anti-DPEAAE rabbit polyclonal antibody (dilution:1/500; catalog number PA1-1748A, Thermo-Fisher Scientific, Waltham, MA), anti-Ki67 (dilution:1/100; clone SP6, Thermo-Fisher Scientific). Alexa fluor 568-conjugated goat anti-rabbit immunoglobulin and goat anti-mouse immunoglobulin (Molecular Probes, Invitrogen) were used as the second antibodies and the nuclei were counterstained and slides were mounted with ProLong Gold Antifade with 4′, 6-diamidino-2-phenylindole (DAPI) mounting medium (Life Technologies, Grand Island, NY). *mT/mG* mouse tissues were embedded in OCT^R^ compound and frozen for cryostat sectioning and slides were mounted with ProLong Gold Antifade with DAPI mounting medium (Life Technologies).

For electron microscopy, eyes were fixed with 2.5% glutaraldehyde plus 4% PFA in 0.2 M sodium cacodylate buffer, pH 7.4 prior to processing and embedding in epoxy resin. Thin sections (85 nm) were stained with osmium tetroxide and viewed with a Phillips CM12/STEM transmission electron microscope (FEI Company, Delmont, PA, USA) equipped with a digital 11-megapixel CCD camera (Gatan, Pleasanton, CA, USA). Lens capsule thickness was measured using Image J software.

### β-galactosidase (β-gal) histochemistry and RNA *in situ* hybridization (ISH)

Whole mount β-gal staining of *Adamts9*^*lacZ/*+^ eyes^32^ was followed by paraffin embedding and 5 μm sections were counterstained with eosin as previously described^32^,^61^. For RNA *in situ* hybridization, (ISH), mouse embryo heads or enucleated eyes were obtained fresh at various ages. Tissues were fixed overnight in 4% PFA/PBS, embedded in paraffin and sectioned (6 μm). ISH was carried out using the RNAscope^®^ technique and custom-designed *Adamts9, Pofut2 and B3glct* probes and a HybEZ™ Oven (RNAscope^®^ 2.0; Advanced Cell Diagnostics, Hayward, CA) according to the manufacturer’s instructions. Target probe binding was disclosed by alkaline phosphatase-staining with Fast Red as substrate and Gill’s hematoxylin as a counterstain. As a negative control, some sections were hybridized with target probe against *DapB,* a bacterial gene encoding dihydrodipicolinate reductase. A target probe directed against ubiquitously expressed *Polr2a* served as a positive control.

### Expression plasmids, cell culture, western blotting and protein purification

hADAMTS9-N-L2 expression plasmid was previously described^38^. hADAMTS9 TSR9-15 was generated by PCR amplification of the encoding cDNA and cloning into pSecTagA (Life Technologies) for in-frame expression with an N-terminal signal peptide and C-terminal myc-His_6_ tag in mammalian cells. PCR was performed using full-length ADAMTS9 cDNA^27^ as the template and primers 5′-AAGGCGCGCCCTCGGTGGAAACCAGTGGAGAACT-3′ and 5′-AACTCGAGTGCAATTCTGGGGTAACTCACAGTT-3′ (restriction enzyme recognition sequences employed for cloning are underlined). Plasmids were transfected into CHO cells (ATCC, Manassas, VA) using Fugene^®^ 6 (Promega, Madison, WI) and stably transfected clones were selected using 500 μg/ml Zeocin (hADAMTS9 TSR9-15, Life Technologies) or 300 μg/ml G418 (hADAMTS9-N-L2). Clones were expanded in DMEM supplemented with 10% fetal bovine serum; conditioned media were tested by western blotting using anti-*myc* mouse monoclonal antibody (clone 9E10, Life Technologies). At confluence, medium was removed and replaced with serum-free medium. After 48 h, the conditioned media were collected and affinity-purified by Ni-NTA chromatography (Qiagen, Germantown, MD) using an AKTA FPLC instrument (GE Healthcare).

### Mass spectral glycoproteomic analysis

The hADAMTS9 constructs purified as described above were reduced, alkylated, and subjected to digestion with Trypsin or Chymotrypsin (Promega). The resulting peptides were analyzed on an Agilent 6340 HPLC-Chip Cube nano LC-Ion trap mass spectrometer as described previously^62^.

### siRNA knockdown experiments

HEK293T cells were purchased (ATCC, Manassas, VA) and co-transfected with negative control (non-targeting siRNA), *POFUT2* or *B3GLCT* siRNA^21^ and plasmid encoding hADAMTS9-N-L2 using Lipofectamine 2000 (Life Technologies) according to manufacturer’s protocol. A plasmid encoding the human IgG heavy chain was co-transfected with siRNAs as a control and IgG analysis in medium and cells was used for normalization of ADAMTS9 levels. 48 hours post-transfection, the cell and media fractions were collected and analyzed by quantitative western blotting using mouse anti-*myc* (9E10) or anti-human IgG (Rockland Immunochemicals, Limerick, PA) on an Odyssey 9120 infrared imaging system (LI-COR, Lincoln, NE).

### Statistics

All data are reported as the mean ± SD. Statistical differences between two groups were analyzed with a 2-tailed Student’s *t* test, assuming a normal distribution. A p value of less than 0.05 was considered statistically significant.

## Additional Information

**How to cite this article**: Dubail, J. *et al*. Impaired ADAMTS9 secretion: A potential mechanism for eye defects in Peters Plus Syndrome. *Sci. Rep.* **6**, 33974; doi: 10.1038/srep33974 (2016).

## Supplementary Material

Supplementary Information

## Figures and Tables

**Table 1:** Summary of eye phenotypes in *Adamts9*^*del/*+^ mice.

Phenotype	Smaller lens	Peters anomaly	Posterior lens extrusions	Disorganized corneal stroma	Lens-cornea adhesion	Iris-cornea adhesion	ciliary body dysplasia	Vacuolar cataract	Persistent nuclei in posterior lens
Newborn	15/17	1/17	16/17	13/17	N/A	N/A	N/A	3/17	1/17
10 day-old	4/6	0/6	6/6	5/6	4/6	5/6	3/6	0/6	2/6
3 week-old	8/10	2/10	10/10	7/10	7/10	7/10	7/10	0/10	2/10

These data are based on observations from H&E stained sections (see also Supplementary Fig. S1b–d). N/A, not available. Note that the fused eyelids in the newborn push the cornea toward the lens, precluding proper visualization of the anterior chamber. Anterior chamber development is not complete until 2–3 weeks, e.g., the ciliary body is not yet developed in the newborn.
