# Supplementary material for: Impaired ADAMTS9 secretion: A potential mechanism for eye defects in Peters Plus Syndrome
Source: Sci Rep. 2016 Sep 30;6:33974. doi: 10.1038/srep33974 (PMC5043182; doi:10.1038/srep33974)
Supplement: Supplementary Information [file srep33974-s1.pdf]

Impaired ADAMTS9 secretion: A potential mechanism for eye defects in Peters Plus Syndrome

Johanne Dubail [1#a], Deepika Vasudevan[2, #b], Lauren W. Wang[1], Sarah E. Earp[1], Michael W. Jenkins[3], Robert S. Haltiwanger[2, #c], and Suneel S. Apte[1]*

Supplementary Information

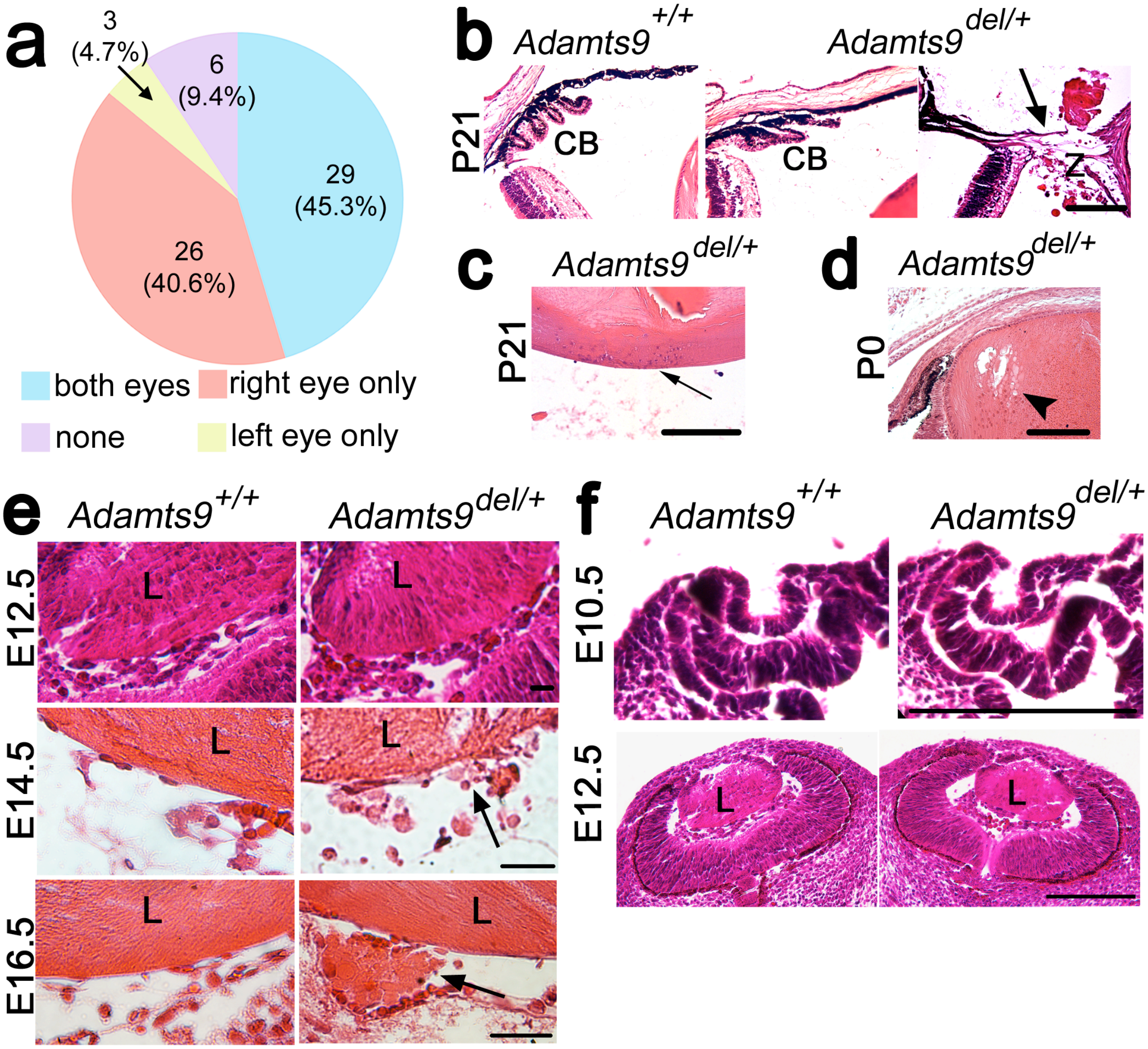

Supplementary Figure S1: *Adamts9* haploinsufficiency led to lens and ciliary body anomalies in postnatal and juvenile eyes. (**a**) Percentage of externally visible ocular defects, specifically corneal opacity, buphthalmos and/or phthisis, in adult *Adamts9*$^{del/+}$ mice (n=64). No ocular anomalies were seen in wild-type littermates. **(b-d)** H&E staining of P21 and P0 *Adamts9*$^{+/+}$ and *Adamts9*$^{del/+}$ eyes demonstrated a hypomorphic and/or dysmorphic ciliary body (CB), presence of lens extrusions into the zonule region (Z,arrow) (**b**), persistence of lens fiber

nuclei in the posterior aspect of the lens (arrow, **c**) and vacuolar cataract (arrowhead, **d)** in some *Adamts9*$^{del/+}$ eyes (Table 1). Scale bar = 100 µm. (**e**) E12.5 and E16.5 *Adamts9*$^{del/+}$ and *Adamts9*$^{+/+}$ eyes were stained by H&E. Abnormal material was observed posterior to the lens in E14.5 and E16.5 *Adamts9*$^{del/+}$ eyes but not in *Adamts9*$^{+/+}$ or in E12.5 *Adamts9*$^{del/+}$ and *Adamts9*$^{+/+}$ eyes. Images are representative of at least 3 eyes analyzed at each time point. L = lens. Scale bar = 25 µm. (**f**) E10.5 and E12.5 *Adamts9*$^{+/+}$ and *Adamts9*$^{del/+}$ eyes stained by H&E were comparable. L = lens. Scale bar = 100 µm.

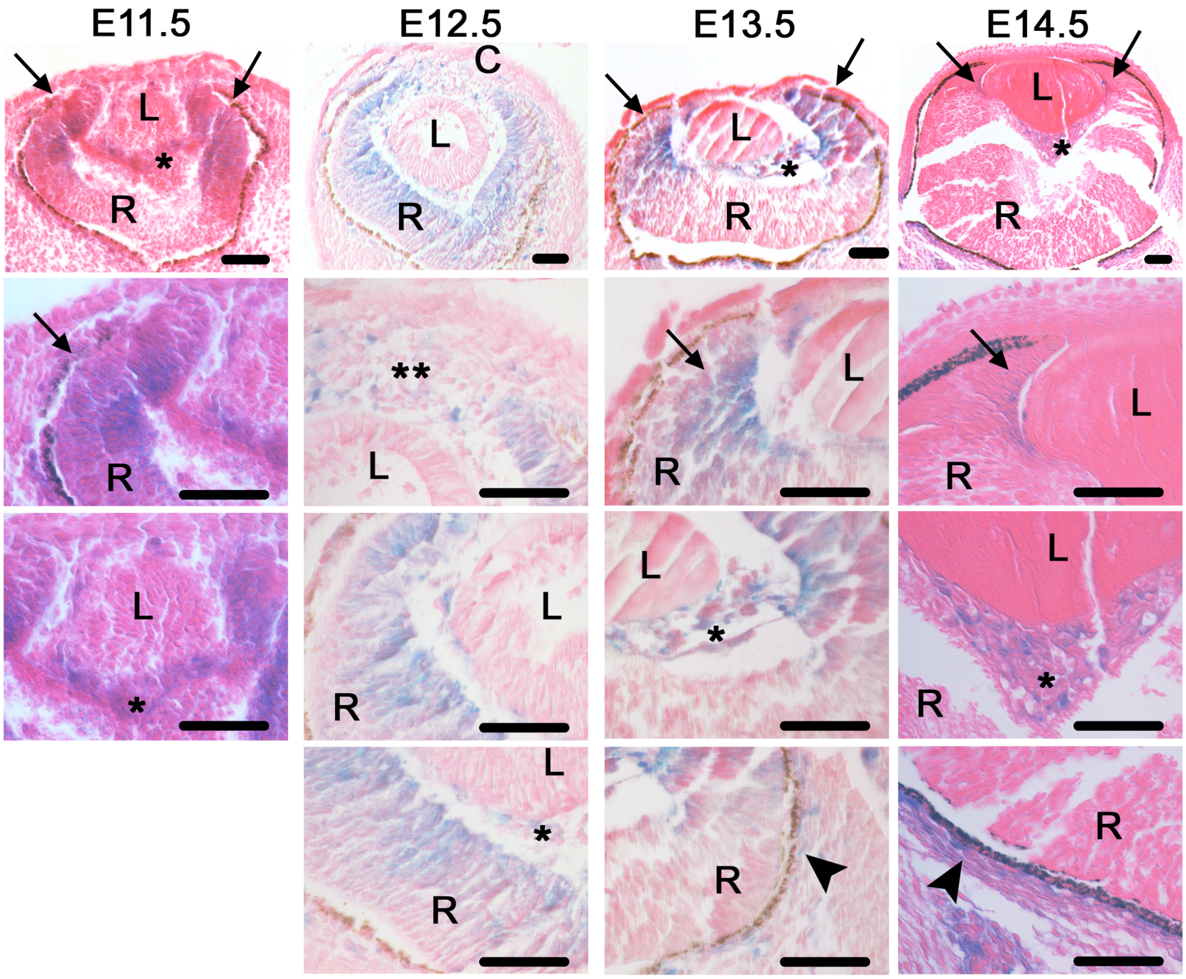

Supplementary Figure S2: Intragenic lacZ expression identifies sites of *Adamts9* mRNA expression during mouse eye development. β-galactosidase staining of E11.5 to E14.5 *Adamts9*$^{LacZ/+}$ eyes confirmed *Adamts9* expression in the anterior pole of the retina (arrows), in the hyaloid vasculature (single asterisk), in some cells anterior to the lens that could arise from the pupillary membrane (double asterisks) and in the choroid (arrowhead). L = lens, R = retina. Scale bar = 50 µm.

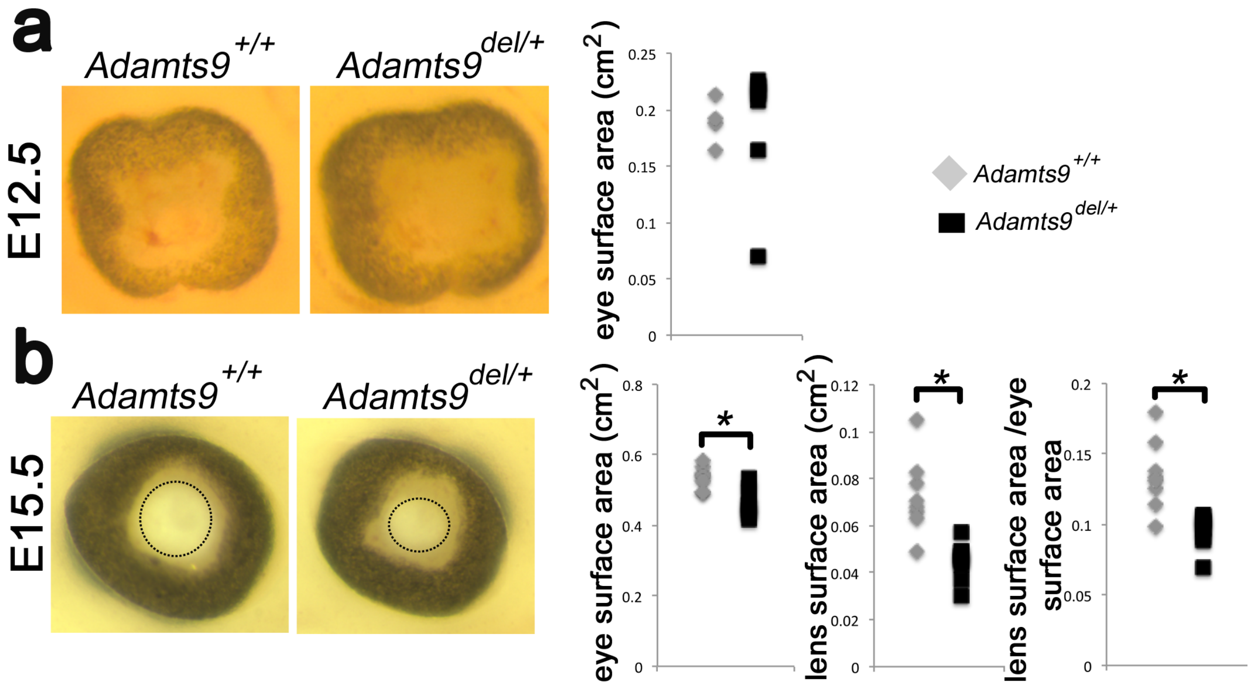

Supplementary Figure S3: *Adamts9* haploinsufficiency leads to reduced eye and lens growth from E15.5 onward. E12.5 (**a**) and E15.5 eyes (**b**) were photographed with a stereomicroscope and the eye or lens area was measured using ImageJ software. *Adamts9*$^{del/+}$ and *Adamts9*$^{+/+}$ eyes were similar in size at E12.5. At E15.5, the eye surface area and lens surface area, appearing white through the pupil after fixation (dotted lines), were significantly reduced in *Adamts9*$^{del/+}$ embryos as compared to *Adamts9*$^{+/+}$ embryos. *: $p<0.01$.

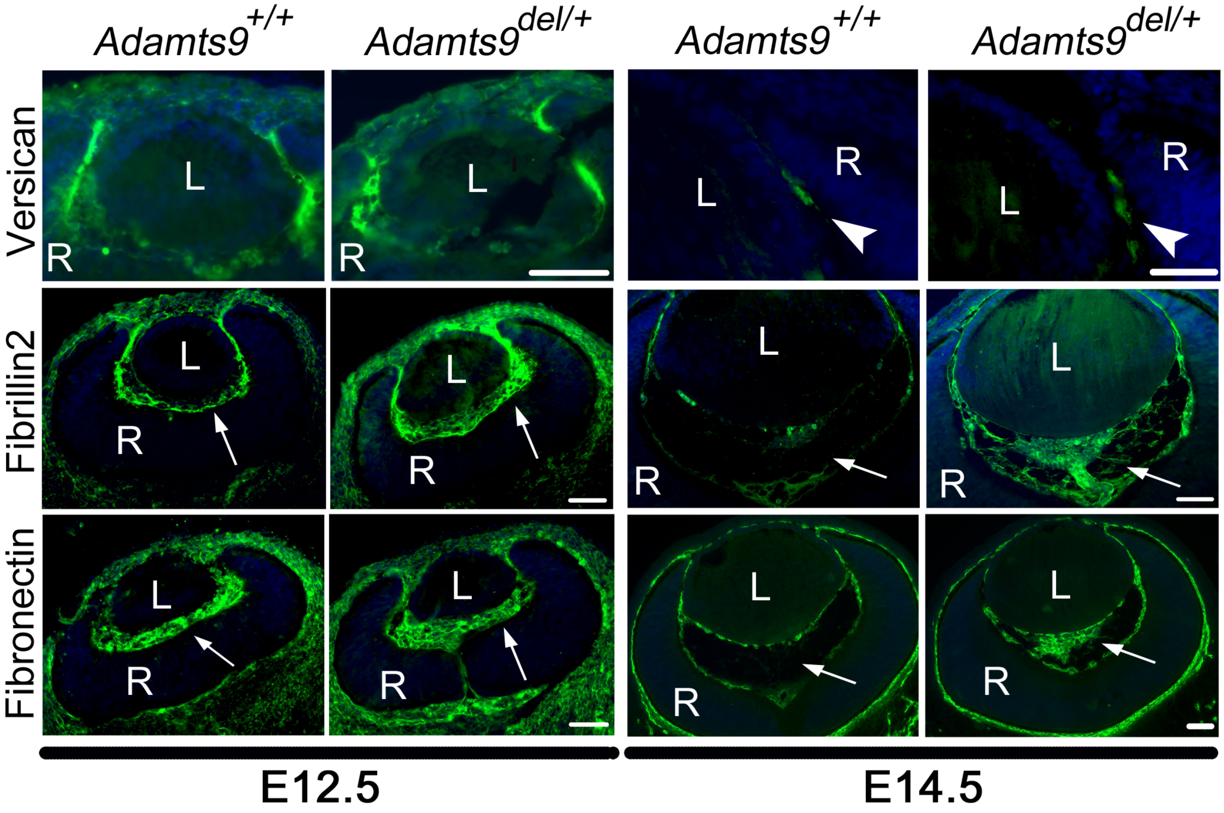

Supplementary Figure S4: Versican immunostaining was unaltered while fibrillin-2 and fibronectin immunostaining was enhanced in *Adamts9$^{del/+}$* eyes. E12.5 and E14.5 *Adamts9$^{del/+}$* and *Adamts9$^{+/+}$* eyes were stained using antibodies against versican, fibrillin-2 or fibronectin (green) and the nuclei were stained with DAPI (blue). Versican immunostaining was similar in *Adamts9$^{del/+}$* and *Adamts9$^{+/+}$* eyes. Fibrillin-2 and fibronectin staining were increased in the hyaloid tissue (arrow) of *Adamts9$^{del/+}$* eyes as compared to *Adamts9$^{+/+}$* eyes at E12.5 and E14.5 (fibrillin-2) or only at E14.5 (fibronectin), respectively. L = lens, R = retina. Scale bar = 25 µm.

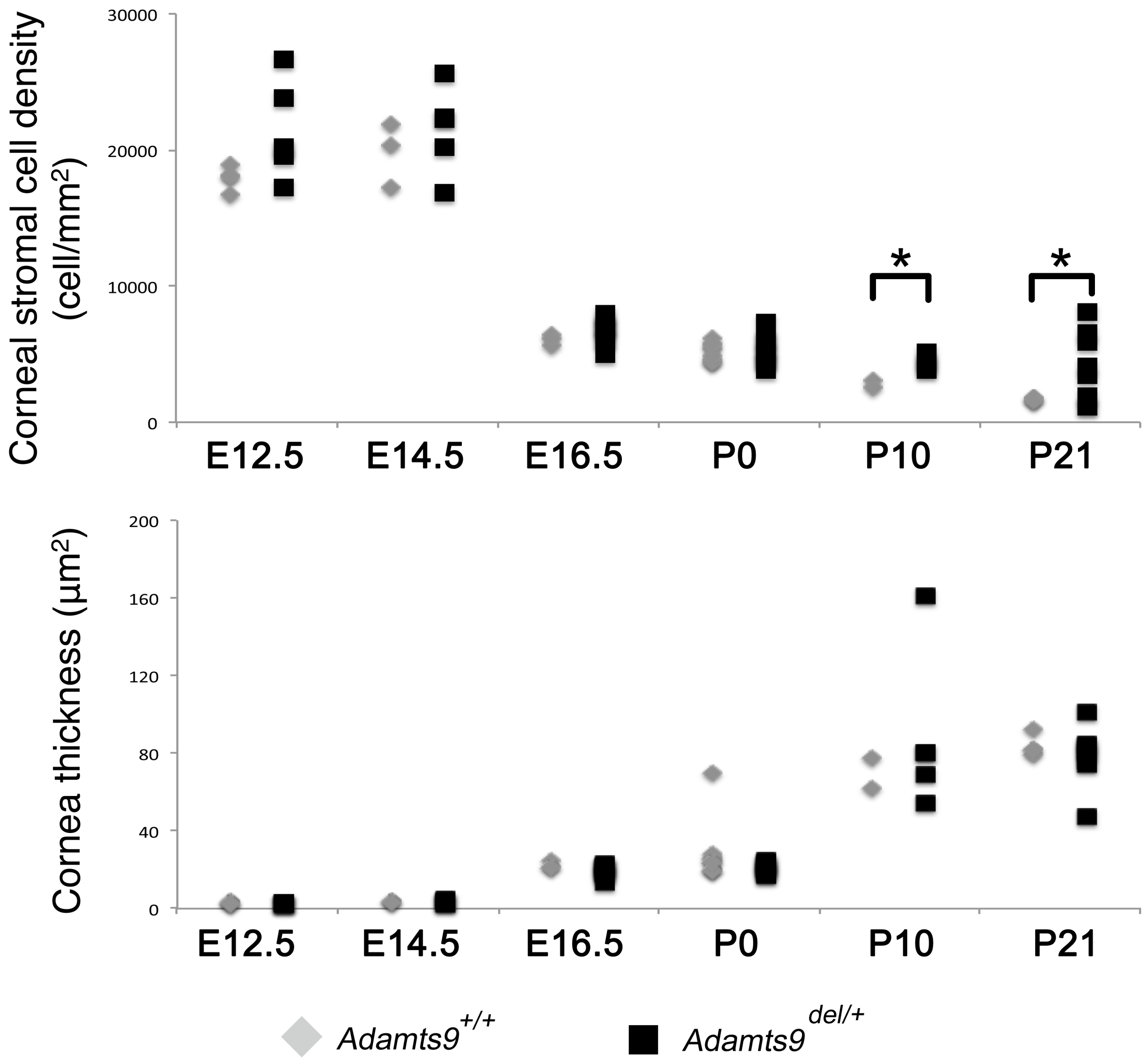

Supplementary Figure S5: Embryonic and newborn cornea had similar cell density and thickness in *Adamts9*$^{del/+}$ and *Adamts9*$^{+/+}$ eyes. H&E stained sections of *Adamts9*$^{del/+}$ and *Adamts9*$^{+/+}$ eyes at different ages were stained with DAPI. Cell density in the corneal stroma and corneal thickness were measured using ImageJ software. *: $p<0.05$.

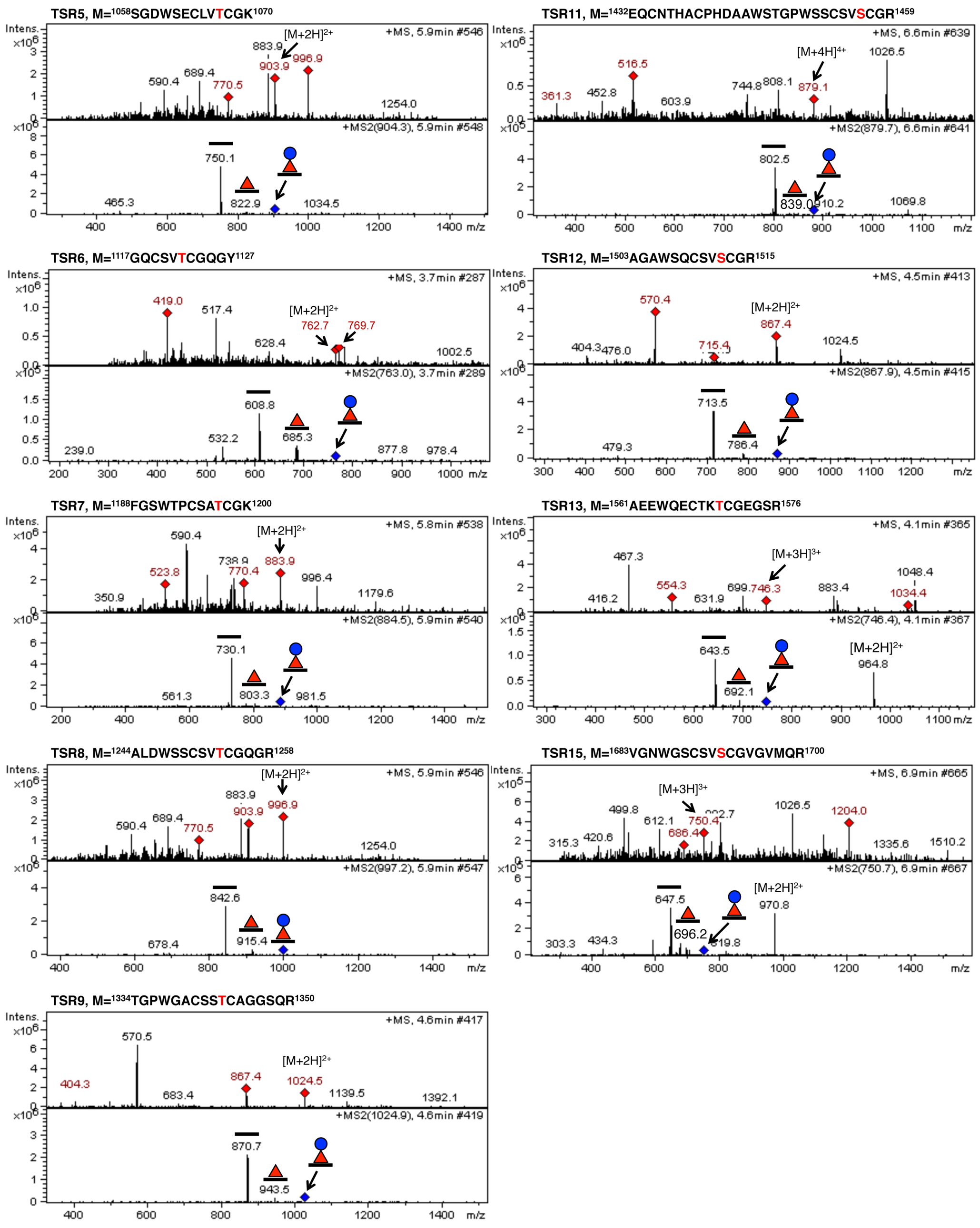

Supplementary Figure S6: TSR5-9, TSR11-13 and TSR15 of ADAMTS9 are modified with Glucoseβ1-3Fucose disaccharide. The top half in each panel shows the mass spectrum at a particular time in the LC run. The parent ion $[M+nH]^{n+}$ corresponding to the peptide from the indicated TSR is indicated by the red diamond. The bottom half of each panel represents the spectrum after collision induced dissociation fragmentation of the parent ion (blue diamond)

which results in the production of an ion whose mass corresponds to that of the unglycosylated peptide (black line) from the indicated TSR. The difference in the mass of this peptide and the parent corresponds to the sequential losses of a hexose (glucose, blue circle) and a deoxyhexose (fucose, red triangle). The sequence of the peptide with the modified amino acid indicated in red is shown at the top of each panel. Table S1 lists the observed masses of the peptides from each TSR mapped.

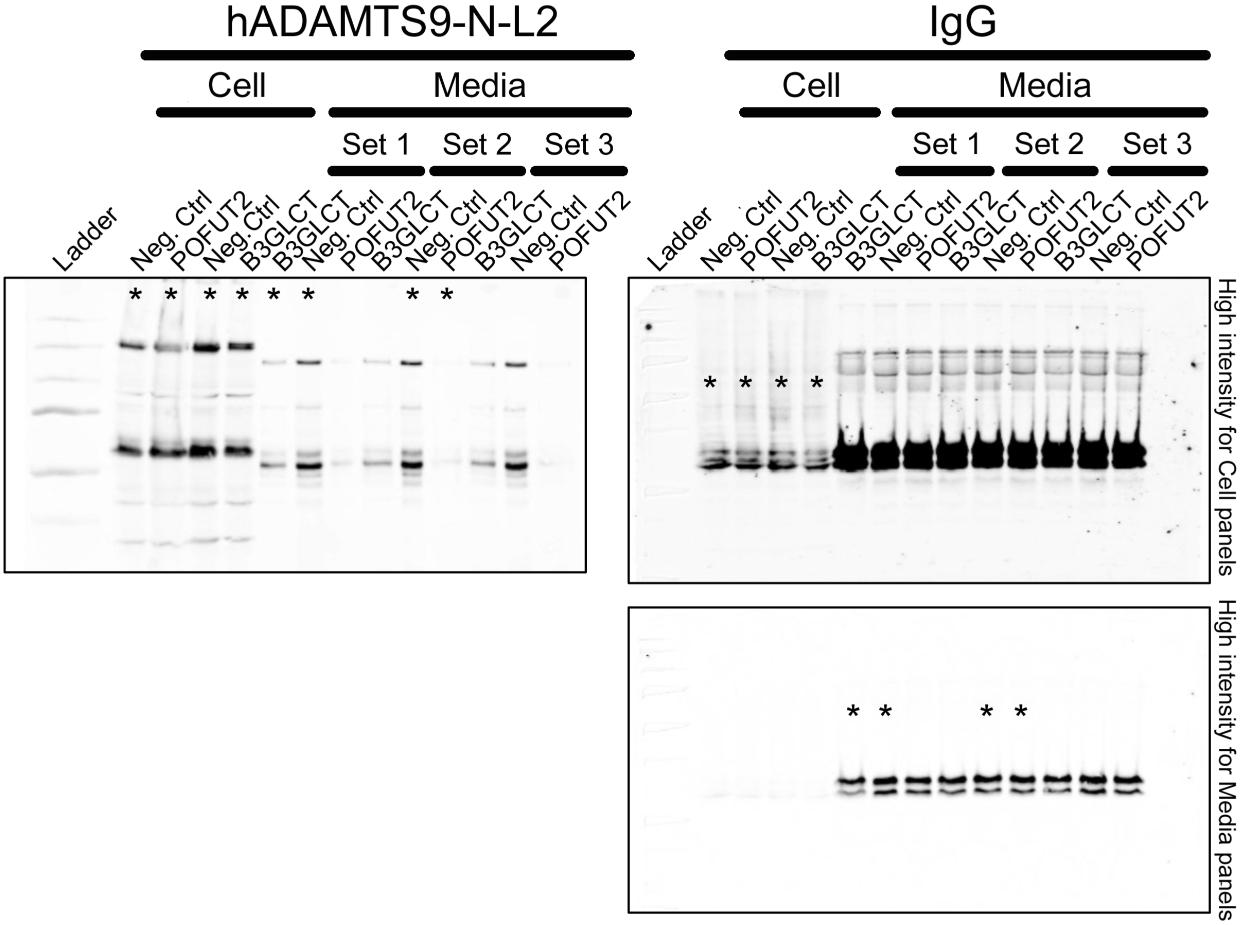

Supplementary Figure S7: Full unedited western blotting gels for Fig. 7. Western blotting analyses were performed as described in Fig. 7. * indicates the lanes used in Fig. 7.

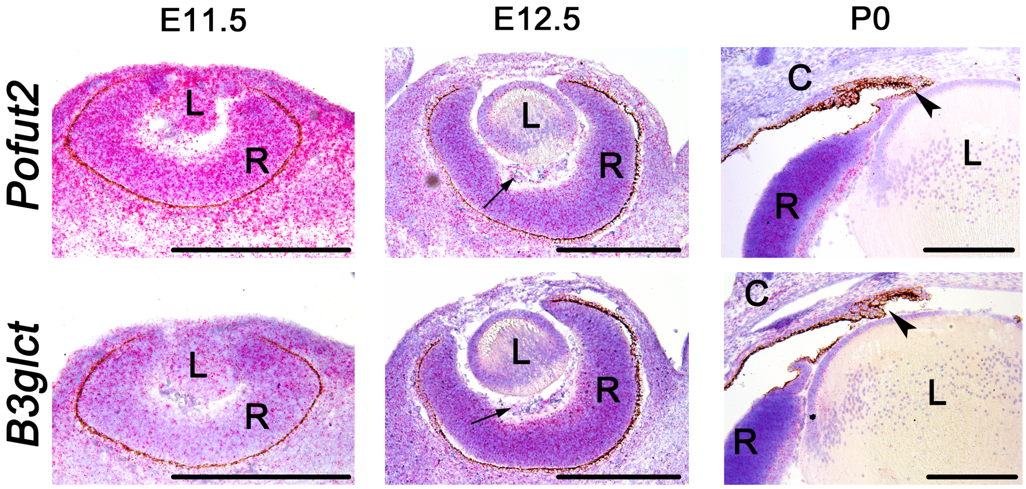

Supplementary Figure S8: ***Pofut2*** **and** ***B3glct*** **mRNA are widely distributed throughout eye development.** The images show ISH using specific *Pofut2* and *B3glct* probes at the indicated ages. Note mRNA expression (red dots) throughout the optic cup/retina (R), lens (L) and cornea (C). The arrows indicated signal in the hyaloid vasculature. The sections were counterstained with hematoxylin. Scale bar = 200 µm.

**TSR**	**Sequence**	**Charge (n)**	**[M+nH]$^{n+}$**	**[M+nH-Hex]$^{n+}$**	**[M+nH-dHex-Hex]$^{n+}$**	**Calculated mass**
5	^{1058}SGDWSECLVTCGK^{1070}	2	904.1	823.1	750.1	750.3
6	^{1117}GQCSVTCGQGY^{1127}	2	763.0	681.8	608.8	609.2
7	^{1188}FGSWTPCSATCGK^{1200}	2	884.1	803.1	730.1	730.3
8	^{1244}ALDWSSCSVTCGQGR^{1258}	2	996.6	915.6	842.6	842.9
9	^{1334}TGPWGACSSTCAGGSQR^{1350}	2	1024.7	943.7	870.7	870.9
11	^{1432}EQCNTHACPHDAAWSTGPWSSCSVSCGR^{1459}	4	879.7	839.0	802.5	804.3
12	^{1503}AGAWSQCSVSCGR^{1515}	2	867.5	786.5	713.5	713.7
13	^{1561}AEEWQECTKTCGEGSR^{1576}	3	746.2	692.2	643.5	643.7
13	^{1561}AEEWQECTKTCGEGSR^{1576}	2	1118.8	1037.8	964.8	965.0
15	^{1683}VGNWGSCSVSCGVGVMQR^{1700}	3	750.2	696.2	647.5	647.7
15	^{1683}VGNWGSCSVSCGVGVMQR^{1700}	2	1124.8	1043.8	970.8	971.1

Supplementary Table S1: Peptides from the MS analysis of ADAMTS9.

Calculated and observed masses of the glycopeptides in Fig. 6 and Supplementary Fig. S6. All peptides were generated from tryptic digests except the peptide from TSR6, which was generated from a chymotryptic digest. Three forms of each peptide were detected: fully modified with fucose and glucose ([M+nH]$^{n+}$), the monosaccharide form modified only with fucose ([M+nH-Hex]$^{n+}$) and unmodified form ([M+nH-dHex-Hex]$^{n+}$). Average mass of the peptide was used for theoretical calculations. Note that for TSRs 13 and 15, two different charge states of the same peptide were observed and the spectra were added to generate the chromatograms in Fig. 6.
